# Supplementary material for: Availability of personal protective equipment and diagnostic and treatment facilities for healthcare workers involved in COVID-19 care: A cross-sectional study in Brazil, Colombia, and Ecuador
Source: PLoS One. 2020 Nov 11;15(11):e0242185. doi: 10.1371/journal.pone.0242185 (PMC7657544; doi:10.1371/journal.pone.0242185)
Supplement: S1 File — Anonymous survey administered to the participants. (DOCX) [file pone.0242185.s001.docx]

P1: On your last call during the care of a suspected respiratory patient, did you change your diagnostic or therapeutic behavior for any of the following reasons?

- P1_1: Unavailability of necessary medication.
- P1_2: Lack of access to non-invasive ventilatory support (oxygen, cannulas, humidifiers, masks).
- P1_3: Lack of access to intensive care or invasive mechanical ventilation (ventilator).
- P1_4: Lack of access to necessary diagnostic imaging tests.
- P1_5: Lack of access to necessary laboratory tests.
- P1_6: I have had the necessary to diagnose/treat patients

P2: On your last shift or day of care, what protective implements were needed to care for suspected respiratory patients? You can choose more than one.

- P2_1: Gloves
- P2_2: Disposable surgical hat
- P2_3: Disposable surgical mask
- P2_4: N95 type mask
- P2_5: Disposable gown
- P2_6: Disposable shoe protectors
- P2_7: Face shield or visor
- P2_8: Clear protective glasses
- P2_9: Special protective closed suit.
- P2_10: Biocidal hydroalcoholic solution
- P2_11: I've had adequate and sufficient PPE

P3: How often are you given personal protective equipment?

- 1: A complete set at shift entrance
- 2: Limited (1-2 of each) within the work area.
- 3: On demand, change of equipment each time I enter or leave a room or environment
- 4: On free demand within the work area
- 5: I do not get delivered; I must bring my own.
- 6: They do not give me any; I do not have any.

P4: Currently this situation has improved, and you have more access to personal protective equipment?

- 1: Yes
- 2: No

P5: If protective equipment is reused, what type of sterilization or cleaning method do you use?

- 1: I do not sterilize, I dispose it.
- 2: I do not sterilize; I keep it and use it again.
- 3: I sterilize it at my own expense in the best possible way.
- 4: I return it at the end of the shift and the medical center takes care of sterilizing it.
- P5_1: Other and comment (codified as 0 in P5)

P6: Have you received any training on how to use personal protective equipment?

For example: In what order should the different items be put on or taken off to maintain security? In what cases should each item be used according to the level of suspicion or the procedures to be carried out?

- 1: Yes, it was enough, and I am comfortable with these concepts.
- 2: Yes, but it was insufficient, and I would like to have clearer information.
- 3: No, I am not familiar with these concepts.

P7: What type of standardized protocols or guidelines for the care of suspected respiratory or COVID positive patients do you use in your facility?

- 1: I do not know, or we don't have any protocols for common use.
- 2: National protocols
- 3: Exclusive private protocols for your center
- 4: Guides of world health organizations, societies, or institutions abroad.

P8: If you have protocols or guidelines, what do you consider to be the main obstacle to their implementation?

- 1: Lack of habit for using protocols or unawareness of it
- 2: It is not possible to follow them due to various limitations (logistics, inputs, etc.)
- 3: They change frequently, and I cannot keep up.
- 4: I have no obstacle to applying the protocols

P9: If you, as a health care worker, have respiratory symptoms, how does the facility handle your possible spread?

- 1: I must get the test and I must continue to work until I have a positive result.
- 2: The institution manages the test, and I must continue to work until I have a positive result.
- 3: The institution manages the test and I wait at home while they give me results.

P10: If you as a healthcare worker have had unprotected exposure to a patient or family member who tests positive for COVID-19, what actions does your facility take?

- 1: Continue to work while you are asymptomatic.
- 2: Preventive isolation at home for 14 days.
- 3: Preventive isolation at home for the time indicated by my center.
- 4: Preventive isolation at home and the center manages access to a test (e.g. PCR, pharyngeal swab)
- 5: Preventive isolation at home, I must get the test on my own.

P11: Which of the following are obstacles to patient care during this crisis? Rank from highest (10 points) to lowest (0 points) in your experience.

- P11_1: Insufficient healthcare workers prior to the pandemic.
- P11_2: Insufficient healthcare workers due to sick leave or exposure to high-risk contacts.
- P11_3: Lack of personal protective equipment.
- P11_4: Lack of medicine.
- P11_5: Insufficient hospitalization room.
- P11_6: Unawareness or absence of COVID-19 care protocols.
- P11_7: Lack of monitors or ventilators.
- P11_8: Lack of serological tests or PCR to identify positive cases.

P12: Currently this situation has now improved and there is more access to adequate equipment and treatment?

- 1: Yes
- 2: No

P13: In your opinion, is there any other obstacle to patient care that has not been mentioned previously?

P14: What is your current position?

- 1: Physician (resident)
- 2: Physician (specialist)
- 3: Nursing Assistant
- 4: Nurse
- 5: Stretcher-bearer
- 6: Respiratory therapist
- 7: Laboratory or Imaging Technician
- P14_1: Other (specify) (codified as 0 in P14)

P15: Place of work:

- 1: Public hospital
- 2: Private hospital
- 3: Primary care
- P15_1: Other (specify) (codified as 0 in P15)

P16: In which area do you mainly work?

It refers to which physical area within the hospital regardless of the service, department, or specialty.

- 1: Emergencies.
- 2: Hospitalization.
- 3: Intermediate or Intensive Care Unit.
- 4: Diagnostic tests.
- 5: Primary care.
