# Supplementary material for: Availability of personal protective equipment and diagnostic and treatment facilities for healthcare workers involved in COVID-19 care: A cross-sectional study in Brazil, Colombia, and Ecuador
Source: PLoS One. 2020 Nov 11;15(11):e0242185. doi: 10.1371/journal.pone.0242185 (PMC7657544; doi:10.1371/journal.pone.0242185)
Supplement: S1 Table — (DOCX) [file pone.0242185.s002.docx]

**S1 Table. Resources for appropriate diagnosis and treatment of COVID-19 patients in several settings.**

|  | **Emergency**  **n= 249/797 (31,6%)** | | **Hospitalization**  **n=214/797 (27,2%)** | | **ICU/Intermediate**  **n=100/797 (12,7%)** | | **Primary care or priority consultations**  **n=199/797 (25,3%)** | | **p value** |
| --- | --- | --- | --- | --- | --- | --- | --- | --- | --- |
|  | **n** | **%** | **n** | **%** | **n** | **%** | **n** | **%** |  |
| **In general, during your previous workdays, during the care of a patient with possible respiratory affection, did you modify your therapeutic or diagnostic behavior for any of the following reasons? You can choose more than one.** | | | | | | | | | |
| **Unavailability of necessary medication** | 76 | 30.5 | 48 | 22.4 | 24 | 24.0 | 57 | 28.6 | 0.2 |
| **Lack of access to non-invasive ventilatory support (oxygen, cannulas, humidifiers, masks)** | 74 | 29.7 | 32 | 15.0 | 13 | 13.0 | -- | -- | <0.001 |
| **Lack of access to intensive care or invasive mechanical ventilation (ventilator)** | 104 | 41.8 | 59 | 27.6 | 19 | 19.0 | -- | -- | <0.001 |
| **Lack of access to necessary diagnostic imaging tests** | 65 | 26.1 | 41 | 19.2 | 17 | 17.0 | 75 | 37.7 | <0.001 |
| **Lack of access to necessary laboratory tests** | 92 | 36.9 | 60 | 28.0 | 29 | 29.0 | 95 | 47.7 | <0.001 |
| **I have had the necessary to diagnose/treat patients** | 50 | 43.5 | 79 | 55.6 | 40 | 59.7 | 50 | 35.5 | 0.001 |

Only physicians and nurses’ responses have been taken into consideration for this analysis (N=797).
