# Supplementary material for: Availability of personal protective equipment and diagnostic and treatment facilities for healthcare workers involved in COVID-19 care: A cross-sectional study in Brazil, Colombia, and Ecuador
Source: PLoS One. 2020 Nov 11;15(11):e0242185. doi: 10.1371/journal.pone.0242185 (PMC7657544; doi:10.1371/journal.pone.0242185)
Supplement: S2 Table — (DOCX) [file pone.0242185.s003.docx]

**S2 Table. Unavailability of personal protective equipment as reported by each professional group.**

|  | **Doctor**  **n= 534/1082 (49,4%)** | | **Nurse**  **n= 263/1082 (24,3%)** | | **Nursing Assistant**  **n= 171/1082 (15,8%)** | | **Others**  **n=114/1082 (10,5%)** | | **p value** |
| --- | --- | --- | --- | --- | --- | --- | --- | --- | --- |
|  | **n** | **%** | **n** | **%** | **n** | **%** | **n** | **%** |  |
| **Gloves** | 64 | 12.0 | 40 | 15.2 | 49 | 28.7 | 21 | 18.4 | <0.001 |
| **Hat** | 86 | 16.1 | 54 | 20.5 | 41 | 24.0 | 27 | 23.7 | 0.06 |
| **Disposable surgical mask** | 99 | 18.5 | 67 | 25.5 | 56 | 32.7 | 30 | 26.3 | 0.001 |
| **N95 type mask** | 304 | 56.9 | 139 | 52.9 | 92 | 53.8 | 65 | 57.0 | 0.7 |
| **Disposable gown** | 184 | 34.5 | 84 | 31.9 | 72 | 41.2 | 51 | 44.7 | 0.03 |
| **Disposable shoe protectors** | 173 | 32.4 | 106 | 40.3 | 71 | 41.5 | 57 | 50.0 | 0.001 |
| **Face shield** | 278 | 52.1 | 136 | 51.7 | 100 | 58.5 | 55 | 48.2 | 0.3 |
| **Clear protective glasses** | 147 | 27.5 | 72 | 27.4 | 53 | 31.0 | 39 | 34.2 | 0.4 |
| **Special protective closed suit** | 331 | 62.0 | 144 | 54.8 | 91 | 53.2 | 77 | 67.5 | 0.02 |
| **Biocidal hydroalcoholic solution** | 26 | 10.8 | 55 | 22.4 | 35 | 20.8 | 16 | 18.2 | 0.005 |
| **I've had adequate and sufficient PPE** | 58 | 24.2 | 50 | 20.4 | 23 | 13.7 | 14 | 15.9 | 0.05 |
