# Supplementary material for: Availability of personal protective equipment and diagnostic and treatment facilities for healthcare workers involved in COVID-19 care: A cross-sectional study in Brazil, Colombia, and Ecuador
Source: PLoS One. 2020 Nov 11;15(11):e0242185. doi: 10.1371/journal.pone.0242185 (PMC7657544; doi:10.1371/journal.pone.0242185)
Supplement: Attachment — Submitted filename: Response to Reviewers.docx [file pone.0242185.s004.docx]

Dear Editor,

We appreciate very much the work done by the reviewers, particularly in this period we assume that is an additional effort as we are well aware of the difficulties arising from the COVID outbreak. Many thanks. Also, we appreciate the suggestions. We are sure they improve this work. We have followed your directions and included a manuscript with tracked changes, and a revised manuscript. In this letter, we comment one by one on these suggestions.

Yours faithfully

The authors

Editorial comments:

1. Please ensure that your manuscript meets PLOS ONE's style requirements, including those for file naming.

We have revised the manuscript in order to meet PLOS One style requirements.

2. Please provide further details on sample size and power calculations. Please provide approximate numbers of health workers (especially doctors and nurses) and treatment facilities for COVID-19 in the 3 countries. Please discuss whether your sample size would be sufficiently representative of your target population.

Further details and information from national and international official statistics sources have been added, and a comment made in the methods section.

3. We suggest you thoroughly copyedit your manuscript for language usage, spelling, and grammar. If you do not know anyone who can help you do this, you may wish to consider employing a professional scientific editing service.

Attached you´ll find Editage certificate. Thank you for the advice provided.

4. We note that you have indicated that data from this study are available upon request.

We have uploaded our data set in an open access file repository:

Mira JJ. PPE, diagnostic and treatment facilities for COVID-19 in South America 2020. doi:10.17605/OSF.IO/4EG8R.

Also, the study is publicly available at ClinicalTrials: NCT04486404

5. Please amend your authorship list in your manuscript file to include author Stefany Pacheco

We have amended our authorship list via the Edit Submission and included author Gloria Pacheco.

Reviewer comments:

1. Please provide a more detailed methodology section. There are lots of items missing.

We have followed the ACCADEMY group guideline for design and conduct of self-administered surveys of clinicians, and we appreciate the advice given by the reviewer. We have made the necessary corrections and provided a more detailed methods section.

2. The columns in each of the tables are different. Please pick one categorisation and stick to it. Where necessary, the other categorisations may be used in the text and listed in the appendix.

We have taken under consideration reviewer advice and included two of the original tables as a supplementary file to be available online. More precisely, former Table 2 and 4. We acknowledge, that having different columns to display information may be confusing but we consider this to be necessary as relevant information is provided. We consider that tables comparing public and private resources available to healthcare workers, differences of Personal Protective Equipment at disposal of healthcare workers who performed high risk of contagion procedures and finally, training for correct use of PPE and use of protocols for healthcare workers to be important, as they align with the recommendations made to managers and policy makers.

3. My preference would be for predominant reporting of descriptive statistics without an emphasis on inference. This survey is simply not designed to answer any inferential questions, though it is very tempting to calculate p values when you have a reasonably large dataset.

We agree with the reviewer and we have made changes to our methods section. Even though we have decided to keep Chi Square analysis in some categories, this could be removed if editorial and reviewers consider it necessary.

4. Please consider listing more of the free text comments in a separate Table- these are very powerful and in my opinion, some of the most important findings from your survey. Please use this platform to help colleagues without a voice be heard.

We cannot agree more with you, relevant information was provided by means of the free text comments, we received a total of 365. We have included Table 5, which lists more extracts and the number of times a similar idea was expressed by respondents.

5. the first paragraph of the discussion should list all the key findings from the study, rather than re-state the aim which has already been done. I would say that the severe PPE shortages, lack of readily available testing and preventative isolation for HCWs and inadequacy of PPE and infection prevention training for HCWs.

We have made the necessary corrections and included key findings in the first paragraph of the discussion.

6. I would also add a “Conclusions” paragraph at the end of the Discussion which again stresses the key messages from the survey and one or two key learning points or future directions.

A “conclusion” paragraph was added at the end of the discussion and also key points for managers and policy makers were included. Hopefully this research can provide feedback and future directions for governments in South America.

7. I understand that due to the language the WHO has been using, you have chosen to separate the groups based on aerosol generating procedures- however, there is increase evidence that simple things like breathing and talking are aerosol generating, and that the aerosol/droplet dichotomy is an oversimplification. Some acknowledgement of this would be appropriate, even if you don’t change your actual methods.

We recognize this dichotomy could be an oversimplification, but we can also agree on that some procedures poses an inherent risk that could only be performed in safe spaces of a hospital. This is why we have included the following phrase in our methods section “This distribution was done considering the increased risk of infection associated with some COVID-19 patient care practices as opposed to others, which normally advise differentiated personal protective equipment needs.” Which is also why, we have considered as important to preserve the table comparing both circumstances, where protocols differ as which PPE are necessary for each of them. We believe, it is also important to communicate how this increased perception of higher risk and exposure of HCW (reported by other studies) can modify their demands. One example of this, is how 60% of participants who do not perform high risk procedures reported as unavailable special protected closed suits, when it is not necessary, according to current protocols.

Minor comments

We have made the necessary corrections and copyedited our manuscript using Editage services.

We have included two objectives in the last paragraph of the introduction.

We have included details about Surveymonkey as the platform used to conduct the study.

More details about the sample and its size has been added to the methods.
