# Supplementary material for: Availability of personal protective equipment and diagnostic and treatment facilities for healthcare workers involved in COVID-19 care: A cross-sectional study in Brazil, Colombia, and Ecuador
Source: PLoS One. 2020 Nov 11;15(11):e0242185. doi: 10.1371/journal.pone.0242185 (PMC7657544; doi:10.1371/journal.pone.0242185)
Supplement: Attachment — Submitted filename: Response to Reviewers.docx [file pone.0242185.s005.docx]

Dear Editor,

We appreciate very much the work done by the reviewer, particularly in this period we assume that is an additional effort as we are well aware of the difficulties arising from the COVID outbreak. Many thanks. Also, we appreciate the suggestions. We are sure they improve this work. We have followed your directions and included a manuscript with tracked changes, and a revised manuscript. In this letter, we comment one by one on these suggestions.

Yours faithfully

The authors

Reviewer comments:

Minor comments

L40: change “it was not randomized” to “sampling was non-random”

We have unified the term to “non-random” across the manuscript.

L56-59: update COVID case numbers and deaths from latest WHO Sitrep

We have updated COVID cases and deaths according to WHO Dashboard, and changed the citation.

L153: change “It was prepared using…” to “We used..” for simplicity

We have included your suggestion. We agree, on simplicity.

Table 3, L212: “Did not performed” should read “Did not perform”

We have corrected the table.

Discussion

L272-273: The first two sentences do not belong here. They should either be deleted or moved later into the discussion. I think you should start with your major findings i.e. “This study has identified severe PPE shortages, insufficient training in infection prevention and PPE usage, and lack of readily available testing and isolation protocols for healthcare workers in Ecuador, Brazil and Colombia…..”

We have modified the first paragraph of the Discussion. Thank you for your advice.

L375: drop this sentence “Otherwise, responsiveness is questioned”.

The sentence has been removed.

L376: “Our study has highlighted some areas that need to be improved **for further waves of COVID-19 and potential future pandemics and epidemics**.”

We have included reviewer suggestion.
