# Supplementary material for: Availability of personal protective equipment and diagnostic and treatment facilities for healthcare workers involved in COVID-19 care: A cross-sectional study in Brazil, Colombia, and Ecuador
Source: PLoS One. 2020 Nov 11;15(11):e0242185. doi: 10.1371/journal.pone.0242185 (PMC7657544; doi:10.1371/journal.pone.0242185)
Supplement: Attachment — Submitted filename: Response to Reviewers.docx [file pone.0242185.s006.docx]

Dear Editor,

We appreciate very much the work done by the reviewers and editorial office, particularly in this period we assume that is an additional effort as we are well aware of the difficulties arising from the COVID outbreak. Many thanks. Also, we appreciate the suggestions. We are sure they improve this work. We have followed your directions and included a manuscript with tracked changes, and a revised manuscript. In this letter, we comment one by one on these suggestions.

Yours faithfully

The authors

Reviewer comments:

Minor comments

To correct minor grammatical errors throughout the manuscript.

We have provided a new version that has been copyedited by Editage and supplied the certificate of editing.

While revising your submission, please upload your figure files to the Preflight Analysis and Conversion Engine (PACE) digital diagnostic tool.

We have followed PLOS ONE requirements and used PACE to ensure that.
